# Structural Basis of Human Astrovirus Entry via FcRn and Antibody Neutralization

**DOI:** 10.1063/4.0001160

**Published:** 2025-10-27

**Authors:** Sashank Agrawal, Ian A Wilson

**Affiliations:** The Scripps Research Institute, La Jolla, California, USA

## Abstract

Human astroviruses (HAstVs) are a common cause of viral gastroenteritis worldwide, especially affecting children, the elderly, and people with weakened immune systems. Despite their global impact, no specific treatments or vaccines exist. Understanding how HAstVs enter human cells is critical for developing new therapies. Recent research identified the neonatal Fc receptor (FcRn) as the key entry receptor for HAstV entry. While FcRn normally helps regulate antibody levels in the body, HAstVs have evolved to use this receptor to infect host cells. However, the precise mechanism by which the virus binds to FcRn has remained unclear.

In this study, we determined the crystal structures of the HAstV spike proteins bound to FcRn to define this interaction at atomic level. We found that the virus attaches to FcRn using a conserved site on the spike, shared across HAstV serotypes. This conserved interaction explains how the virus infects cells and highlights a target for antiviral development. We also investigated how neutralizing antibodies block infection. Most published antibodies prevent HAstV from binding FcRn by physically blocking the interaction, rather than by competing for the same site. These insights provide important information for designing vaccines and antibody-based therapies. Overall, our findings clarify the molecular mechanism of HAstV entry and identify a promising target for broad antiviral strategies. This work lays the foundation for developing new interventions to prevent and treat HAstV infections, addressing a significant unmet medical need.

